# A Serious Game Approach to Improve Food Behavior in Families—A Pilot Study

**DOI:** 10.3390/nu12051415

**Published:** 2020-05-14

**Authors:** Sigrid Skouw, Anja Suldrup, Annemarie Olsen

**Affiliations:** Food Design and Consumer Behavior Section, Department of Food Science, University of Copenhagen, Rolighedsvej 26, 1958 Frederiksberg C, Denmark; ssn@food.ku.dk (S.S.); clg487@alumni.ku.dk (A.S.)

**Keywords:** serious game, gamification, eating behavior, food neophobia, willingness to taste

## Abstract

The objective of this pilot study was to investigate the effect of a specially developed serious game to improve food behavior in families with children aged 5–13 years using mixed methods. Fourteen families were randomized into a game-group and a non-game-group and divided into age groups (game-children (GC), game-parents (GP), non-game-children (nGC), and non-game-parents (nGP)). The families completed a baseline test, a three-week intervention period with or without a game element, and a follow-up test. Qualitative results showed a positive change in food behavior in all families. Quantitative results mainly showed an effect in food neophobia as a decrease was seen in all groups; however, it was only significant (*p* < 0.05) in three groups (GP, nGC, nGP). No changes were seen in willingness to taste, and only limited changes in liking and number of words used to describe the stimuli. In conclusion, qualitative results showed positive change in the children’s food behavior in most families, indicating a positive effect of performing tastings and tasks together as a family—regardless of the presence of a game element. However, this was not as clear in the quantitative data, indicating that current quantitative tools are less suited to measure complex concepts like willingness to taste.

## 1. Background

Low intake of fruit and vegetables (F&V) was according to WHO among the top 10 leading risk factor causes of death in middle- and high-income countries and among 6 diet-related risks of disability-adjusted life years in 2004 [1]. Surveys from 2005 [2] and 2014 [3] showed F&V intake among European 11-year-old children to be below the recommended levels of 400 g/day [4].

Issanchou and Nicklaus [5] put together a conceptual framework showing a number of different concepts determining children’s food choice, one of these being experience and social influence from parents and peers. Genetics will affect children’s sensory perceptions, and parenting style will further be determining preferences, choices, and intake [5]. This has also been shown in experimental research, like a recent review of different strategies to change children’s eating behavior [6]. Parental control and using rewards/instrumental feeding was shown to largely impact eating behavior both positively and negatively. Examples of such strategies are availability of food in the household, restriction of the amount of food a child is allowed to eat, and use of rewards to get children to eat particular foods [6].

A report on vegetable consumption in Denmark showed intake to be limited to only a few types of vegetables such as carrot, onion, and tomato [7]. The most limiting factors of vegetable purchase in Danish families were found to be the lack of ideas on how to use and to get children to eat different and new vegetables [7]. Children’s limited food choices are also a challenge in other countries. For instance, a survey from Uruguay found similar results of low variety of vegetable intake and low liking, and some vegetables were never offered to the children due to either parents not eating them themselves or not knowing how to prepare them [8]. To meet the national recommendations for fruit and vegetable intake and to prevent picky eating and food neophobia (reluctance to eat new foods [9]), these limiting factors should be addressed. Encouraging families to approach novel or disliked F&V in a more explorative manner may reduce these limitations, e.g., through sensory exploration and involvement [6], and increase in F&V intake.

The use of game elements to change eating behavior has gained more attention over the last two decades [10,11,12]. Games created with the intention of developing skills and knowledge are classified as *serious games*. Serious games were initially defined by Abt [13] as games that “… *have an explicit and carefully thought-out educational purpose and are not intended to be played primarily for amusement. This does not mean that serious games are not, or should not be, entertaining*”. Playing games is usually associated with fun social interactions of a competitive nature and is driven by both intrinsic and extrinsic motivation [14,15], providing a hands-on approach. The latter has been found more effective in increasing vegetable consumption in children compared to educational programs [6]. Thus, specially designed games might be useful tools for motivating and encouraging exploration of a variety of foods, including that of F&V, and to further promote a change in eating behavior.

Games have demonstrated potential for increasing children’s F&V consumption [16,17], while studies on the effect of games on adults are scarce and show only little or no effect [18,19]. Investigation into the effect of games on families does not exist to the knowledge of the authors of this study, constituting a gap in knowledge. This gap is particularly interesting as efforts made to change eating behavior have been found to be more efficient when directed at the family level rather than at the individual level [20], since parental food habits is one of the most important determinants of children’s food choice and behavior [21].

Thus, the aim of this pilot study was to investigate if a specially developed serious game could improve food behavior in relation to fruit and vegetables in families with children aged 5 to 13 years. Food behavior was investigated through measures related to the game content: food neophobia and willingness to taste, food vocabulary used to describe F&V, and qualitative measures.

## 2. Material and Methods

### 2.1. Recruitment and Randomization

Sixteen families were recruited through social media and a newsletter shared by the project Taste for Life (a research and communication collaboration of scientists in Denmark with focus on taste, www.taste-for-life.org) to participate in the pilot study. Inclusion criteria were no F&V allergies and address in or around the area of Copenhagen for logistic reasons. Most participating families consisted of two children and two parents. Some families contained one or two children in the target age group and one child outside of the target age group, who participated in the game but not in the tests. The families were randomly assigned to either a game group or a non-game. Two families (one game and one non-game family) dropped out of the study before the baseline test; one for unknown reasons and another due to illness. The game-group and non-game-group each contained seven families at the beginning of the intervention. The study complied with the Helsinki declaration. After reviewing the study protocol, the study was found not to require ethical approval (j.nr. 19007287). The data collection and handling plan was approved by the institutional GDPR office (j.nr.: 514-0120/19-5000). Parents gave written, informed consent on behalf of themselves and their children, and children agreed to participate and for data to be used for scientific publications.

### 2.2. Intervention Material

Developing a game, which unites fitting motivators, a fitting social situation, and mere exposure to novel or disliked foods through sensory interactions, have the potential to be a successful strategy to encourage food exploration and possibly change eating behavior in families.

A serious game was developed for the purpose of this study and was called *The Kingdom of Taste*. The game is played by up to five players and is composed of:

1. One game board with 30 boxes marked on it of which 23 contains a task or action to be done;
2. 88 food cards (with names and pictures of F&V, works as point cards);
3. 5 colored game pieces;
4. One die;
5. One booklet containing an illustrated backstory and the rules;
6. Parental instructions with examples/suggestions on how to solve the different types of tasks;
7. 6 cups with lids (used to contain and hide taste samples of F&V to be used in the game).

Six different F&V are to be used during the game. The F&V are cut into appropriate pieces, one for each player, and placed in the six cups and covered with the lids. The lids were included to add an additional element of surprise and excitement for the players, as they would not see which F&V they were to taste, before landing on a taste task. This could potentially change the level of arousal and the participant’s optimal complexity of foods, as described by Dember and Earl [22], before uncovering and thereby affect willingness to taste the hidden F&V.

**Figure 1:**
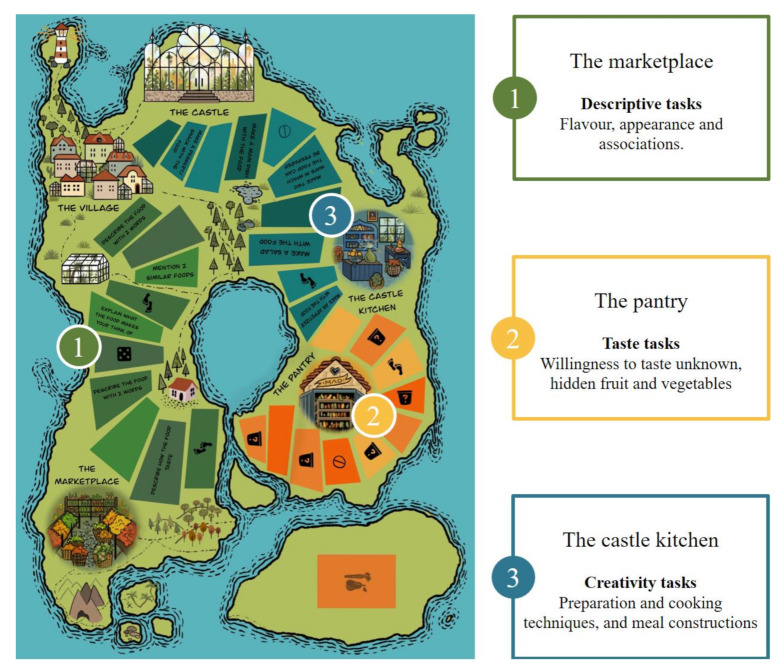
The Kingdom of Taste and an overview of the three zones and their distinctive tasks.

The game is typically played by 3–5 players (1–3 children and 2 parents from the participating families) and takes 30–60 min. The board game is centered around a story of a chef who has forgotten to purchase F&V for a dinner party at the castle. The chef asks the players to help him collect as many F&V (point cards) as possible on their way from the village to the castle. To collect F&V, the players have to solve different tasks present on the game board. The tasks fall within three categories: (1) descriptive tasks where F&V are to be described with regard to flavor, appearance, and associations; (2) taste tasks where the players has the opportunity of tasting up to six different and unknown F&V; and (3) creativity tasks related to preparation, cooking techniques, and construction of meals. The tasks are represented on the game board as three distinctive zones as shown in Figure 1. The tasks are created to increase familiarity of a large variety of F&V, both through descriptive tasks and through tastings. Mere exposure to the F&V through pictures, words, and tastings could potentially increase affection of these [23]. Creative meal planning is a part of the game in the last zone and as the game is finalized by each player composing a three-course meal with his/her collected F&V card (points). Sparking exploration and interest in meal composition could inspire players to bring this creativity to the kitchen and further affect food behavior. The game aimed at obtaining a suitable level of difficulty for the target group, in accordance with the Theory of Flow, which describes how the relationship between skill level and posed challenges needs to be balanced to achieve a state of flow; i.e., when the challenge a person is faced with is not too difficult nor too easy to solve [24,25]. Before the pilot test, the game was tested by five families with children aged 4 to 9 years and one school class with students aged 11 to 12 years. The families received all necessary materials (except F&V, which they were to provide themselves with the possibility of receiving compensation for their purchases) and a questionnaire with questions regarding the game elements, age group, entertainment, etc., to be filled out after having tested the game. The game was modified according to this feedback.

The game was used as intervention material for the game-families.

The non-game-families were provided with a representative selection of the three categories of tasks present on the board game, including tastings of F&V, but without the game context. The non-game material was comprised of a sheet of task instructions, 30 food cards (with names of the F&V and no pictures), one parental instruction with examples/suggestions on how to solve the tasks, and containers without lids for taste samples. Lids were not included in order to limit any game element that could create additional excitement during a task.

### 2.3. Study Design

The study timeline consisted of a baseline test, a three-week intervention period, and a follow-up test. The families were instructed to perform their designated assignments at home once a week during the three-week intervention period; i.e., the game-families were to play the serious game, and the non-game-families were to perform similar tasks without the game element a total of three times. F&V for the home assignments were delivered to all families (both game and non-game families) once a week during the intervention period, at their home address.

Table 1 provides an overview of the taste samples used at the home assignments. The F&V for taste samples were chosen based on the theory of *Zone of Proximal Development* [26], as this theory has recently been used to explain flavor preference development in children [27]. The theory of the Zone of Proximal Development is originally a model with three levels (Zone of Actual Development, Zone of Proximal Development, and Zone of Insurmountable Difficulty) describing children’s cognitive development as a result of social interaction between individuals with different skill levels [26]. In a food context, The Zone of Actual Development represents foods that are familiar, liked, and considered to be safe to eat for the child on his/her own, whereas the Zone of Proximal Development represents foods that are considered exiting, unknown, and associated with some degree of uncertainty, which the child is only able or willing to taste under adult guidance and support [27]. F&V choices aimed at having two F&V belonging to the Zone of Actual Development (upper two rows in Table 1) and four F&V in the Zone of Proximal Development (lower four rows in Table 1).

At the end of the follow-up session, all families received a 500 DKK (75 USD) gift card of their choice as a thank-you-gift for their participation in the study, and the non-game-families further received a *Kingdom of Taste* game.

### 2.4. Questionnaire Design and Test Protocol

Baseline and follow-up tests were performed at the university with a maximum of two families present in the same room at a time placed in far ends of the room, never mixing families from different intervention groups. Each test took approximately one hour.

Mixed methods were applied for the data collection of this study in order to obtain a fuller insight into the intervention effects, as a combination of quantitative and qualitative methods can provide insights that may be missed when only using one of them [28]. The choice of using mixed methods was further based on the expectation that food behavior is a complex concept, which is possibly difficult to measure by current quantitative tools. The questionnaire developed for the baseline test consisted of three quantitative parts: (1) A Danish version of the six-item version of the original food neophobia scale (FNS) [29], first used in [30]; (2) a box for describing the presented F&V (a measure of food vocabulary, single words only); and (3) willingness to taste the presented F&V (yes/no), including liking measured on a 7-point hedonic smiley scale and stating familiarity and frequency of consumption to provide an idea of the level of preliminary knowledge about the F&V used in the tests. The questionnaire was to be filled out individually, though the younger children had the opportunity to receive assistance either from one of the two first authors of this article or their parents.

The follow-up test consisted of the same quantitative tasks as the baseline test to measure change during the intervention period, but further included qualitative questions for the parents to answer. The qualitative questionnaire consisted of open-ended questions regarding observed changes in food behavior at home during the intervention period; changes in willingness to taste, how they discussed F&V, and changes in the children’s engagement with F&V.

The F&V used at the baseline and the follow-up test were the same; banana, carrot, broccoli, papaya, prune, and caper berry, which were chosen based on the same considerations as the taste samples for the home assignments. The order of the F&V was randomized and varied between all the families at the two test sessions. The F&V were placed on two plates, one plate with taste samples and another plate with the F&V in its true shape (uncut, except for the broccoli and papaya which were cut in half), for the participants to use as a visual tool when answering the questions. The F&V were presented one at a time. Additional materials present were pens, paper plates, napkins, crispbread, cups, and water.

### 2.5. Data Analysis

All statistical data analyses were conducted using R-studio statistical free software (version 1.1.456, Boston, United States) [31]. Graphs were made using Microsoft Office Excel (2016) and Microsoft Office PowerPoint (2016).

The participants were divided into groups according to treatment and age group: game-children (GC), non-game-children (nGC), game-parents (GP), and non-game-parents (nGP). Baseline differences between GC vs. nGC and GP vs. nGP were tested for by conducting a Mann–Whitney U test for age, gender, food neophobia, liking, and word count, and Fisher’s exact test was used to test for baseline differences in willingness to taste.

A McNemar test tests if two response variables are significantly different from each other within a study sample and was used for testing significant differences in willingness to taste (yes/no) between baseline and follow-up within each of the four groups. A linear mixed model tested for differences within each of the four groups between baseline and follow-up in the FNS score, liking, and word count. The collected words were both analyzed as total word count as well as count within the word-categories *hedonic, descriptive*, and *other*. The model was further used to test the difference in change found between the treatments in both age groups (GC vs. nGC and GP vs. nGP) for the same measures. Residuals of the linear model not following a normal distribution were transformed by a log-transformation.

A Cronbach’s alpha test was run on the FNS scores in each of the two age groups to test reliability. The test was run on data from baseline and follow-up test separately.

The qualitative feedback collected at the follow-up test was analyzed by using a combination of pre-set and emerging codes (willingness to taste, food language, food engagement, game related) with individual emerging sub-codes.

## 3. Results

Two non-game-families dropped out of the study just before the follow-up test; one due to illness and another due to scheduling issues. Seven game-families and five non-game-families completed the follow-up test. Three children who participated in the baseline test did not participate in the follow-up test due to illness. A total of 12 families and 39 participants completed the entire study; 22 in the game-group and 17 in the non-game-group. Table 2 shows the age and gender distribution in the two groups. No differences were found in age or gender distribution when comparing the treatments in both age groups.

### 3.1. Quantitative Measures

#### 3.1.1. Food Neophobia, Willingness to Taste, and Liking

**Figure 2:**
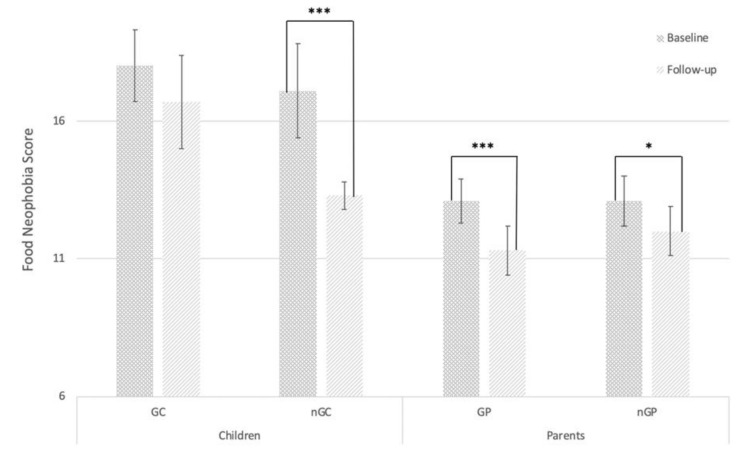
Mean (±SEM) range of food neophobia scale (FNS) score at baseline and follow up. Abbreviations: Game-children (GC), non-game-children (nGC), game-parents (GP), non-game-parents (nGP). Significance level estimated by a linear mixed model. Significance levels: *** *p* < 0.001, ** *p* < 0.01, * *p* < 0.05.

No significant differences were found at baseline between the treatments in both age groups in FNS score. All groups showed a decrease in FNS score from baseline to follow-up test, but significant reductions in FNS score were only found in nGC, GP, nGP but not in GC, as shown in Figure 2. Of the 10 GC, seven showed a decrease, one remained unchanged and two showed an increase in food neophobia at follow-up.

Cronbach’s Alpha was calculated for the FNS score of the children and the parents separately. At baseline α = 0.64 for both the children and parents, and at follow-up α = 0.8 and 0.7 for children and adults, respectively. These sizes indicate consistency.

No significant difference in willingness to taste was found between treatments and age groups at baseline or between baseline and follow-up in any of the groups, and only minor and scattered changes in liking between baseline and follow-up were observed as seen in Table 3.

#### 3.1.2. Food Vocabulary Used by Families to Describe F&V

Food vocabulary was measured through counting the number of single words used to describe a given F&V, and categorized them in one of three word categories: *Hedonic* (e.g., “delicious”), *descriptive* (e.g., “green”) or *other* (e.g., “monkey”). Only a few significant changes in number of words used to describe the presented F&V was found. When a significant change was present, it was characterized as an increase in word count in the non-game-group and a decrease in the game-group, with no general tendency of specific word groups increasing or decreasing more than others (Table S1). The changes in words were not specifically connected to any of the three word-categories hedonic, descriptive, or other.

### 3.2. Qualitative Measures

#### 3.2.1. Perceived Change in Food Behavior

Food behavior was measured as willingness to taste, food language, and food engagement in the qualitative questionnaire. All 12 families reported an increase in willingness to taste in the qualitative questionnaire. Six families, four game and two non-game, expressed that their food language had changed over the course of the intervention period. Eight families, five game and three non-game, indicated that they have been having food-related conversations during the intervention period. Nine families, five game and four non-game, reported an increase in the children’s food engagement on one or more parameters: increased interest in food, cooking, or meal planning. When summarizing the qualitative results, parents reported improved food behavior independently on the presence of a game element. 

#### 3.2.2. Motivational Effect of the Game Element

Six of the seven game-families commented on the use of a game to increase willingness to taste. Four of them reported how the game/competitive element in the game increased their children’s willingness to taste the F&V in the home assignment, as expressed by one mother: “*The game/competitive element in the game caused our children to not want to lose and* (they) *tasted almost everything the last couple of weeks*”. Two of the game families further commented that the taste tasks were the most exciting part of the game. The mother of one game-family wrote: “*During the home assignments it was obvious that the children were looking forward to tasting the food, and that the best part was when someone landed on a taste task*”. Two game families further reported how their children had requested to play the game during the intervention period.

## 4. Discussion

### 4.1. Food Neophobia and Willingness to Taste

Several studies have found food neophobia to decrease from childhood to adulthood [32,33,34]. Based on this knowledge, the lower level of food neophobia in the parents in comparison to the children was expected.

FNS have been used to measure the effect of sensory education on food behavior, showing lower scores after intervention [35,36] but not always significantly [35]. All four groups in this study showed a decrease in FNS after intervention either significantly (GP, nGP, and nGP, *p* < 0.05) or non-significantly (GC) corresponding with existing literature. Whether the change in FNS scores is persistent is unknown, as long-term effects were not investigated in this study, but the change indicates the existence of subjective perceptions of change among the participants, at least during the intervention period. This perception of change may result from the participants’ own observations of behavioral changes, such as increased courage to try new foods or being less particular about which foods to eat during and after the completion of the home assignments. This indicates that continuous use of the home assignments may potentially change food neophobia persistently, as a result of increasing willingness to try foods and thereby increase exposure to disliked and novel foods, potentially giving rise to the effect of mere exposure [23].

All 12 families reported that they had experienced an increase in willingness to taste, independent of treatment, supporting the decrease in FNS scores and indicating that the serious game did not provide an additional effect over the tasks performed without the game element.

The game-families generally ascribed the increase in willingness to taste to the competitive element of the game due to its motivational effect. Overcoming a personal boundary of tasting something unknown or novel might function as an intrinsic motivation for the participants, caused by feelings of satisfaction and joy of self-accomplishments, or due to enjoyment of playing the game [14]. As the game further gives rise to extrinsic motivation through the possibility of earning points, winning, and receiving feedback and praise from other players, the participants might further be more motivated to engage [14]. The combination of both intrinsic and extrinsic motivations thereby seems to have resulted in a high degree of willingness to taste during the home assignments in the game-families. The game-families only reported a positive outcome of using extrinsic motivation to get their children to taste the F&V in the game, but previous studies have indicated negative outcomes, as reviewed by DeCosta et al. [6]. Using extrinsic motivation, may have an undermining effect on intrinsic motivation [37], as e.g., parental prompting and restriction of food intake have been found to cause children to override their internal cues of hunger, satiety, and pleasure [6], which could lead to overeating or other negative consequences. These findings could indicate potential negative consequences of using a game to improve food behavior and willingness to taste although this was not indicated by the results of this study. A potential explanation could be the more positive type of extrinsic motivation found in the game compared to a normal eating situation, such as the possibility of gaining rewards (point cards, praises, and cheers) and the wish to win.

In the non-game-families, several of the parents reported that they were impressed by how many of the novel F&V their children were willing to taste during the home assignment. The intrinsic motivation of self-accomplishment might likewise have occurred in the non-game-families during the home assignment. The surprise from the parents’ side, that their children were willing to taste the large variety of F&V presented, could also be an example of the discrepancy in expected pickiness between children and parents found by the Danish Agriculture and Food Council [38], as parents perceive their children to be pickier than the children themselves are. The children could have been willing to taste such F&V before the intervention period but may not have been served it due to the parents’ expectations of their refusal to taste it. In a review by Scaglioni et al. [21], parental food habits were shown to be one of the most important determinants of children’s food choice and behavior. Together with the gap between parental and child beliefs about picky eating, this could be part of the reason why the F&V consumption of European children does not meet the recommendations [2,3]. The children are to a large extent limited in their F&V selection to what is available in the kitchen at home—and what is available and served at home might be limited to what the parents believe to be what their children like [7]. The F&V used in this study were selected to be a mix of well-known and novel stimuli and thereby expose the families to F&V other than what they usually eat. Simply tasting and experiencing novel F&V could be a way to enlighten parents of their children’s higher willingness to taste and eat new F&V than what they believe and thereby be a motivation to incorporate such new foods in the kitchen, which will likely lead to increased F&V intake.

The taste samples used in the game-group were kept a secret until the point of tasting (hidden in a container with lid), whereas the samples were visible for the non-game-families from the beginning of their home assignment (placed in a container without lid). This additional element of secrecy present in the game-families’ assignment may have increased the level of arousal before the reveal of the F&V [22]. Even if the F&V hidden were well-known or at least known to some degree, the participants would not know before opening the container in which they were hidden. The anticipation of what was hidden could possibly increase the arousal, in contrast to the non-game-group where the participants were able to see the F&V before engaging in tasting.

No changes in willingness to taste were found, which does not align with the reduction in FNS scores and the qualitative perception of increased willingness to taste. This lack of difference in willingness to taste could be caused by the high willingness present at the baseline test. Using willingness to taste might thereby not be the best measure for investigating a change in courage to taste different foods, when the stimuli is mostly well-known F&V and when the participants are not neophobic. Other studies have found varying effects of using willingness to taste, ranging from positive effects [36] and temporary effects [35] to no change [39]. This line of thought was recently shared by Olsen [40], who suggests that the focus in this area of research is too narrow and could benefit from using a broader specter of outcome measures, including qualitative ones. If this is the case, the varying effects of sensory education on willingness to taste [35,36,39] may be explained by the inadequacy of the measurement approach rather than the sensory education itself. Other approaches have been made in an attempt to develop a behavioral food neophobia measure for children, such as using wiliness to taste where the children were to taste an unknown food based on their own previous indication of willingness [35], and correlating it with the FNS, but the correlations found between the two tests were generally weak [35,41]. The poor correlation between the FNS and the behavioral food neophobia tests indicates that the two tests may measure two different things. More research into how to effectively measure these, which are considered closely related concepts, is required in order to perform this kind of studies.

The difficulties of using willingness to taste as a behavioral measure of food neophobia may indicate that willingness to taste is a far more complex concept than simply a yes/no question. It may be assumed that different levels of novelty and resistance towards certain foods exist, which may mean that the action of tasting a novel food is rejected but does not necessarily mean that other forms of interactions with the novel food are rejected. Such other interactions could potentially result in willingness to taste at a later time point because of increased familiarity [42]. This speculation is supported by the findings of Dazeley and Houston-Price [43] and Coulthard and Sealy [10], who both found that non-taste sensory interaction increased children’s tasting afterwards.

### 4.2. Food Vocabulary Used by Families to Describe F&V

The limited changes in word count when describing the F&V are in accordance with previous studies, where 11- to 13-year old children showed a decrease in number of words used to describe bread in both an intervention and control group after sensory education [44]. Mustonen et al. [44] expected the lower number of words to be partly due to restlessness in the children during the follow-up test, which was also observed in this study in several children. Likewise, several parents showed signs of restlessness and appeared to use less time on this task at the follow-up test. As the baseline and follow-up test questionnaires were identical, an explanation could be that the writing tasks at follow-up test perhaps were perceived as tedious and not as exciting and fun as tasting the unknown F&V. This speculation is backed up by the qualitative data, where the tasting part was described by two families to be the best part of the game. The tendency to an increase in word count in the control groups and decrease in the intervention groups could indicate that the game element was interfering with the descriptive tasks. The game players might have been eager to move on to other parts of the game that they found more fun, as indicated by some of the game-families, as described in Section 4.4. Focus of the non-game-families, on the other hand, may have been more on the task itself, as there were no game elements. Due to the simpler nature and the limited number of tasks (each participant only having to answer four questions per session), more effort may have been put into solving the non-game. If the outcome is to achieve a more nuanced food language through increasing vocabulary and ability to describe F&V, better results might be achieved by completing the tasks without a game element.

### 4.3. Qualitative Measures

The qualitative feedback received form the parents showed a positive improvement in food behavior in both groups, indicating that the specific tasks (describing, tasting, and being creative with F&V) present in both the game and non-game assignments possibly are sufficient on their own to improve food behavior. It is not possible to tell which element of the home assignment caused the improvements seen in both groups or if it was a collaborative effect.

Both the game tasks and the non-game tasks caused the families to designate time specifically to explore F&V by sensory and mental interactions together which may be a contributing factor to why a positive effect was found in both groups. The positive effect of designating time to these types of tasks has also been found in other studies [10,43,45]. The social situation can also affect willingness to taste through listening to others’ reflections and expectations and observing their behavior towards the F&V [46,47].

### 4.4. Can Serious Games Improve Food Behaviour in Families?

The results of this pilot study do not show any additional effect of using a serious game to improve food behavior in families compared to performing similar non-game tasks, despite the fact that other studies [10,48] have found an effect of physical games on vegetable consumption in children, indicating a potential effect of games. As this pilot study failed to show an effect, further research into this specific segment and topic should be done in order to fully understand the possible outcomes. Conducting a similar study on a larger scale with increased intervention time is recommended in order to investigate if long-term use of the intervention materials would show additional differences between the groups. The additional motivators [14,15] of the game may give rise to continuous use of the game as indicated in the qualitative feedback where the GC requested to play the game during the intervention period. Such continuous use would result in continuous exposure to F&V and here potentially facilitate long-term effects through mere exposure [23]. On the contrary, the non-game tasks may become more tedious in the long run due to fewer motivators. This speculation was substantiated in the qualitative feedback by several of the parents in the game group, mentioning the game as an important motivator. Two game-families reported how their children had requested to play the game again, substantiating the motivational effects of a game.

On the other hand, it is still worth considering the possibility of a long-term effect of the non-game tasks as well, as they are less confined to a specific situation (a game situation), and therefore may be more easily incorporated into a busy lifestyle. Although playing *The Kingdom of Taste* has the potential to be more motivating over time, the non-game tasks may become integrated into the family’s food habits more effortlessly and thereby constitute easy and accessible tools to introduce novel foods. If elements of the home assignments are adopted as new habits in everyday meal situations, rather than requiring the family to set aside time specifically to do the tasks in the format used in this study, the positive effect may occur more automatically and effortlessly [49]. The results of the simple tasks performed by the non-game-families in this study are an example of how little effort it takes to improve food behavior. It seems that it is a matter of making a habit of tasting and discussing ingredients, flavors, etc., of F&V together in an explorative manner—leading to a continuous introduction to and integration of novel F&V, which could cause a shift in food choice and behavior. Ultimately, this could result in overcoming the limiting factors faced by parents of introducing novel vegetables [7]. These speculations on turning the elements of the home assignments into everyday habits are not substantiated by the collected data, as long-term effects were not investigated, causing a need for further research.

### 4.5. Strengths and Limitations

The use of mixed methods in this study constituted a strength, as the qualitative data provided insights that would otherwise not have been discovered through the quantitative data, markedly changing the discussion and conclusion of the study. The major limitation to the study was the small sample size, which over the course of the intervention period was reduced from 49 to 39 individuals, and as mentioned previously, it would be valuable to conduct a similar study with increased sample size and time span. Another limitation was that the families were recruited from a small geographical area in or around Copenhagen, potentially limiting the diversity of family lifestyles and social and environmental surroundings. Copenhagen has a large percentage of people with high educational levels compared to other parts of Denmark, which is expectedly reflected in this study sample and can have caused a bias as educational levels have been found to correlate to diets and health. Furthermore, as participating families were recruited through social media and newsletters by Taste for Life, they can be expected to have a higher interest in food than the general population, which implies that results may not extrapolate to all families. Different family compositions (varying from two parents and three children to two parents and one child) and children’s age will likely have an impact on the effect of the game concerning level of help provided and adaptation of tasks. Another limitation arises from the younger children being able to receive help from their parents or the experimenter to fill in the test questionnaire. Although parents were requested not to help their children with anything other than writing, it is uncertain whether patents fully complied with the instructions, and they may also have suggested responses to their children. This could potentially cause a difference between children able to write by themselves and children not able to write by themselves. It is not possible to know if the children, who requested to play the game again, did it to gain attention from their parents or if it was because they wanted to play the game. Due to the limited period of time available to complete the study, it was not possible to measure if the effect of the intervention was persistent over time. It is recommended that future studies contain a control group with no tasks or tasting of F&V to be able to measure any possible differences in effect between using the game and not doing any tasks.

## 5. Conclusions

In conclusion, most families reported improved food behavior towards F&V in the children—regardless of the presence of a serious game. This indicates that designating time as a family to taste and discuss attributes and handling of F&V is enough to improve food behavior. However, the quantitative results were not as clear, as most measures showed no or limited change. A decrease in food neophobia score was seen in all four groups; however, it was only significant for the parental groups and the non-game-children, indicating no difference between the treatment groups. The lack of complete alignment between the quantitative and qualitative results raises the question of whether current quantitative measures are capable of truly reflecting concepts as complex as willingness to taste and food behavior. Based on these findings, conducting a similar study of larger scale to investigate if these results are persistent is recommended. Results of such a study could be used to consider if future research in this area should initially focus on developing new and better ways of measuring the complex concepts within this field of study by adopting a broader approach of both quantitative and qualitative measures.

## 6. Future Perspectives

Based on the discussion of appropriate measures to investigate a change in food behavior, a better approach may be to evaluate the journey towards willingness to taste, instead of the end point (tasting). The authors of this article therefore suggest that *food exploration* could serve as a new concept, which through both quantitative and qualitative measures allows the assessment of many different ways of interacting with novel food. Exploring foods can take place in several ways, both as a sensory interaction (tactile, olfactory, visual, auditory, gustatory) or as a mental interaction (e.g., using one’s imagination to compose a meal, associating one food with another food, memory, etc.). Examples of exploring a novel or disliked food could for instance be a sensory-based description of a food based on flavor and appearance or a combination of sensory and mental interaction, as seen in the study by Coulthard and Sealy [10], where pre-school children created pictures using F&V. The concept of food exploration acknowledges the existence of different levels of novelty and resistance towards certain foods. If a person is not comfortable with tasting a novel food, he or she might be comfortable with interacting with the food in other ways. Engaging in non-taste sensory or mental interaction with foods might give rise to willingness to taste at a later time point.

## Figures and Tables

**Table 1:** Overview of the taste samples used for the home assignments.

Week 1	Week 2	Week 3
Apple	Banana	Cucumber
Carrot	Tomato	Pear
Fennel	Passion fruit	Dried goji berries
Water chestnut ^a^	Enoki mushrooms	Bamboo shoots ^a^
Jerusalem artichoke	Onion sprouts	Turmeric root
Green olives ^a^	Nashi pear	Cherimoya

^a^ Conserved in brine.

**Table 2:** Age and gender distribution of participants who completed both baseline and follow up test.

	Game Group (*n* = 22)	Non-Game Group (*n* = 17)
**Children**		
Number of children (n)	10	8
Age (mean ± SEM, range)	9 ± 0.9 (5–13)	8 ± 0.6 (6–10)
Gender (female, n (%))	5 (50%)	4 (50%)
**Parents**		
Number of parents (n)	12	9
Age (mean ± SEM, range)	40 ± 0.1 (35–47)	38 ± 0.8 (35–41)
Gender (female, n (%))	6 (50%)	5 (55.6%)

Children (*n* = 18) and parents (*n* = 21).

**Table 3:** Mean (SEM) of liking of the six fruit and vegetables (F&V) at baseline and follow-up (1 = super bad; 2 = really bad; 3 = bad; 4 = neither good nor bad; 5 = good; 6 = really good; 7 = super good).

		Children					Parents				
		GC		nGC		Diff. p^b^	GP		nGP		Diff. p^b^
		Mean (SEM)	p^a^	Mean (SEM)	p^a^		Mean (SEM)	p^a^	Mean (SEM)	p^a^	
Carrot	Baseline Follow-up	5.6 (0.3) 5.5 (0.4)		6.1 (0.4) 5.8 (0.5)			6.3 (0.2) 5.9 (0.3)		5.9 (0.4) 5.6 (0-3)		
Banana	Baseline Follow-up	6.3 (0.3) 6.3 (0.3)		6.7 (0.2) 5.5 (0.9)			6.3 (0.3) 5.8 (0.3)	***	6.4 (0.4) 6.3 (0.3)		
Broccoli	Baseline Follow-up	4.7 (0.4) 5.0 (0.4)		4.3 (1.0) 4.5 (0.8)			5.6 (0.2) 5.7 (0.3)		5.4 (0.2) 5.4 (0.5)		
Papaya	Baseline Follow-up	3.6 (0.3) 4.3 (0.4)	*	2.9 (0.3) 4.4 (0.5)	***		4.0 (0.3) 4.6 (0.3)		2.9 (0.4) 4.9 (0.3)	***	
Prune	Baseline Follow-up	5.0 (0.5) 5.0 (0.6)		5.2 (0.7) 5.1 (0.6)			5.1 (0.4) 5.0 (0.3)		4.8 (0.5) 5.4 (0.4)	*	*
Caper berry	Baseline Follow-up	3.1 (1.0) 4.2 (1.2)		2.3 (1.0) 2.6 (0.8)	*		4.5 (0.4) 4.7 (0.4)		3.6 (0.6) 4.0 (0.7)		

Significance level estimated by a linear mixed model. Abbreviations: Game-children (GC), non-game-children (nGC), game-parents (GP), non-game-parents (nGP). Significance levels: *** *p* < 0.001, ** *p* < 0.01, * *p* < 0.05. ^a^ p-value shows significant level of change from baseline to follow-up in the groups. ^b^ p-value shows significant level of difference in change from baseline to follow-up between CG and nCG and between GP and nGP.

## Supplementary Materials

The following are available online at https://www.mdpi.com/2072-6643/12/5/1415/s1. Table S1: Mean (SEM) number of words used to describe the six F&V in the baseline and follow-up test and significance in difference for the treatment and age groups.
