# Supplementary material for: A Serious Game Approach to Improve Food Behavior in Families—A Pilot Study
Source: Nutrients. 2020 May 14;12(5):1415. doi: 10.3390/nu12051415 (PMC7284603; doi:10.3390/nu12051415)
Supplement: Supplementary file 1 [file nutrients-12-01415-s001.pdf]

Supplementary files

Table 1: Mean (SEM) number of words used to describe the six F&V in the baseline and follow-up test, and significance in difference for the treatment and age groups.

		Children					Parents				
		GC		nGC			GP		nGP		
		Mean (SEM)	p[a]	Mean (SEM)	p[a]	Diff. p[b]	Mean (SEM)	p[a]	Mean (SEM)	p[a]	Diff. p[b]
**Carrot**											
Total	Baseline	3.7 (0.6)		3.9 (0.4)			4.9 (0.6)	*	4.9 (0.4)		*
	Follow-up	2.9 (0.3)		4.1 (0.3)			3.8 (0.5)		5.5 (0.8)		
Hedonic	Baseline	0.2 (0.1)		0.3 (0.2)			0.3 (0.1)		0.3 (0.2)		
	Follow-up	0.2 (0.1)		0.0 (0.0)			0.0 (0.0)		0.0 (0.0)		
Descriptive	Baseline	2.7 (0.5)		3.3 (0.4)		*	3.2 (0.3)		3.4 (0.8)		
	Follow-up	2.4 (0.4)		4.0 (0.3)			2.6 (0.3)		4.4 (0.8)		
Other	Baseline	0.8 (0.4)	*	0.4 (0.4)			1.5 (0.5)		1.3 (0.8)		
	Follow-up	0.3 (0.2)		0.1 (0.1)			1.3 (0.3)		1.1 (1.0)		
**Banana**											
Total	Baseline	4.2 (0.9)		4.0 (0.4)			5.1 (0.8)		5.1 (0.7)		
	Follow-up	3.5 (0.5)		4.3 (0.4)			3.8 (0.4)		4.6 (0.6)		
Hedonic	Baseline	0.3 (0.2)		0.5 (0.2)			0.4 (0.1)		0.4 (0.2)		
	Follow-up	0.1 (0.1)		0.0 (0.0)			0.3 (0.1)		0.0 (0.0)		
Descriptive	Baseline	2.8 (0.6)		2.8 (0.4)	*		3.0 (0.6)		4.0 (0.9)		
	Follow-up	2.9 (0.5)		4.0 (0.5)			2.8 (0.3)		3.9 (0.6)		
Other	Baseline	1.1 (0.6)		0.8 (0.4)			1.7 (0.4)		0.8 (0.5)		
	Follow-up	0.5 (0.2)		0.3 (0.2)			0.7 (0.1)		0.8 (0.3)		
**Broccoli**											
Total	Baseline	3.1 (0.8)		3.4 (0.3)			4.7 (0.5)		4.6 (0.6)		
	Follow-up	3.2 (0.5)		3.5 (0.4)			3.9 (0.5)		4.3 (0.6)		
Hedonic	Baseline	0.4 (0.2)		0.4 (0.2)			0.3 (0.1)		0.9 (0.3)		
	Follow-up	0.2 (0.1)		0.0 (0.0)			0.0 (0.0)		0.1 (0.1)		
Descriptive	Baseline	1.5 (0.3)		2.4 (0.3)			2.8 (0.6)		2.1 (0.4)		
	Follow-up	1.4 (0.3)		3.0 (0.5)			2.3 (0.3)		2.8 (0.5)		
Other	Baseline	1.2 (0.6)		0.6 (0.2)			1.5 (0.4)		1.6 (0.5)		
	Follow-up	1.6 (0.4)		0.5 (0.2)			1.6 (0.3)		1.4 (0.6)		
**Papaya**											
Total	Baseline	3.8 (0.8)		3.5 (0.6)			4.6 (0.5)	**	4.3 (0.9)	***	***
	Follow-up	2.8 (0.6)		4.1 (0.7)			3.3 (0.5)		4.9 (0.6)		
Hedonic	Baseline	0.1 (0.1)		0.5 (0.2)			0.6 (0.3)		0.8 (0.4)		
	Follow-up	0.1 (0.1)		0.0 (0.0)			0.2 (0.1)		0.4 (0.2)		
Descriptive	Baseline	2.6 (0.5)		2.8 (0.5)		*	2.8 (0.5)		2.3 (1.0)	**	**
	Follow-up	1.7 (0.4)		3.6 (0.7)			2.3 (0.4)		4.1 (0.7)		
Other	Baseline	1.1 (0.4)		0.3 (0.2)			1.3 (0.2)		1.3 (0.5)		
	Follow-up	1.0 (0.4)		0.5 (0.3)			0.8 (0.2)		0.4 (0.2)		
**Prune**											
Total	Baseline	2.9 (0.3)		3.5 (0.6)			4.4 (0.5)		4.1 (0.5)		
	Follow-up	3.6 (0.6)		3.9 (0.4)			4.3 (0.4)		4.5 (0.4)		
Hedonic	Baseline	0.0 (0.0)		0.1 (0.1)			0.1 (0.1)		0.4 (0.3)		
	Follow-up	0.1 (0.1)		0.0 (0.0)			0.0 (0.0)		0.3 (0.2)		
Descriptive	Baseline	2.3 (0.3)		2.6 (0.6)			3.2 (0.5)		2.8 (0.5)		
	Follow-up	2.3 (0.7)		3.1 (0.4)			3.4 (0.4)		3.8 (0.4)		
Other	Baseline	0.6 (0.3)		0.8 (0.3)			1.2 (0.3)		1.0 (0.4)		
	Follow-up	0.9 (0.3)		0.8 (0.2)			0.9 (0.3)		0.5 (0.3)		
**Caper berry**											
Total	Baseline	3.1 (0.3)		3.3 (0.4)			3.7 (0.6)		3.4 (0.3)		
	Follow-up	2.5 (0.4)		3.4 (0.5)			3.7 (0.5)		4.3 (0.5)		
Hedonic	Baseline	0.1 (0.1)		0.4 (0.2)			0.5 (0.2)		0.3 (0.2)		
	Follow-up	0.2 (0.1)		0.1 (0.1)			0.3 (0.1)		0.1 (0.1)		
Descriptive	Baseline	2.3 (0.3)		2.3 (0.5)		*	2.5 (0.4)		2.8 (0.4)		
	Follow-up	1.7 (0.4)		3.0 (0.6)			3.1 (0.5)		3.8 (0.6)		
Other	Baseline	0.7 (0.2)		0.6 (0.4)			0.7 (0.3)		0.4 (0.3)		
	Follow-up	0.6 (0.3)		0.3 (0.3)			0.3 (0.1)		0.4 (0.3)		

Significance level estimated by a linear mixed model. Abbreviations: Game-children (GC), non-game-children (nGC), game-parents (GP), non-game-parents (nGP). Significance levels: $p < 0.001$ ***, $p < 0.01$ **, $p < 0.05$.

[a]p-value show significant level of change between baseline and follow-up in the groups.

[b]p-value show significant level of difference in change between the groups.
